# The relationship between self-stigma and quality of life in long-term hospitalized patients with schizophrenia: a cross-sectional study

**DOI:** 10.3389/fpsyt.2024.1366030

**Published:** 2024-06-05

**Authors:** Fuquan Liu, Hu Deng, Na Hu, Wenqian Huang, Hong Wang, Lin Liu, Jiabao Chai, Ying Li

**Affiliations:** ^1^ Beijing Huilongguan Hospital, Peking University Huilongguan Clinical Medical School, Beijing, China; ^2^ Department of Psychosomatic Medicine, Beijing Children’s Hospital, Capital Medical University, National Center for Children Healthy, Beijing, China

**Keywords:** schizophrenia, self-stigma, quality of life, self-esteem, coping strategies

## Abstract

**Objective:**

To investigate self-stigma’s influence on schizophrenia patients’ quality of life and its mediated impact by various factors.

**Methods:**

This study adopted a cross-sectional design and randomly selected 170 hospitalized patients with schizophrenia for evaluation. The assessment tools included the Positive and Negative Syndrome Scale (PANSS), Internalized Stigma of Mental Illness Scale (ISMI), Schizophrenia Quality of Life Scale (SQLS), and Coping Questionnaire for Schizophrenia Patients (CQSP), among others. Correlation analysis, regression analysis, and mediation analysis were used to test the correlation and mediation effects.

**Results:**

Self-stigma had a significant impact on quality of life (T = 8.13, *p* = 0.00). When self-stigma is used as a mediator, the problem-solving factor in coping strategies has an indirect effect on quality of life, which is significant (AB = -0.16, *P* = 0.02), while the avoidance factor in coping strategies has a direct effect on quality of life, which is significant (C’ = 0.54, *p* < 0.001), and an indirect effect, which is also significant (AB = 0.25, *p* < 0.001).

**Conclusion:**

The study highlights the significant impact of self-stigma on the quality of life of schizophrenia patients, emphasizing the crucial roles of self-esteem and coping strategies. These findings suggest clinical interventions to improve quality of life should focus on reducing self-stigma, especially enhancing self-esteem and promoting adaptive coping strategies. By addressing these factors, we can better support the mental health and well-being of those with schizophrenia, offering an effective approach to rehabilitation.

## 1 Background

Mental illness stigma (1) refers to the stigmatization of people with mental illness, which can be divided into self-stigma (2) and public stigma (3). The former refers to the stigmatizing attitude of patients toward themselves, while the latter refers to the personal experiences of discrimination and unfair treatment that patients face from society and the public (4). In addition to the fear and avoidance of social and public discrimination, including stereotypes, prejudices, and discrimination, self-stigma also involves a sense of shame that hinders people from talking about their experiences and seeking help (5). Schizophrenia is defined as a severe mental illness that generally requires long-term social and functional rehabilitation (6, 7). Nevertheless, the internalization of stigma related to schizophrenia is linked to a poorer prognosis and heightened suicidal tendencies (8). Self-stigma is an important obstacle to the social and functional rehabilitation of patients with schizophrenia (9). Stigma experienced by individuals with schizophrenia leads to a lack of self-esteem (10) and continuous self-deprecation and limits normal life and social interactions, ultimately resulting in adverse consequences such as avoidance of social situations, depression, suicide, and a decreased quality of life (11, 12). Therefore, it is important to focus on exploring self-stigma in schizophrenia and its impact on the quality of life of those affected.

Studies on self-stigma in the context of schizophrenia have revealed a direct correlation: as the severity of the mental illness increases and the number of hospitalizations rises, the intensity of self-stigma also escalates (13). Self-stigma leads to decreased self-esteem, reduced hope, restricted social interaction, and decreased compliance (14). It increases self-isolation and reluctance to accept treatment and other support, exacerbating the recurrence of mental illness and seriously affecting the recovery process (15). These proximal effects can also have potential distal consequences, such as hindering the pursuit of life goals, reducing community engagement, impeding social relationships and social support, and ultimately affecting quality of life (16). A meta-analysis of 54 studies conducted by Gerlinger et al. showed that perceived or experienced shame is associated with more severe depressive symptoms, greater social anxiety and avoidance behaviors, low self-esteem, poorer social functioning, and overall lower quality of life (17). The self-stigma of patients with schizophrenia is closely related to their quality of life (18), but there is still insufficient research on long-term hospitalized patients. The harm of self-stigma manifests itself in various ways, as it weakens self-esteem and self-worth and undermines the hope and optimism of achieving goals (19, 20).

At the same time, self-stigma is influenced by various factors. Ritsher et al. developed a five-factor scale for self-stigma, including self-esteem, psychological resilience, coping strategies, social support, etc. (21). These factors have different degrees of influence on self-stigma, but a systematic exploration of the factors affecting self-stigma is lacking. Moreover, whether these influencing factors affect quality of life via self-stigma also needs further investigation. In China, long-term hospitalized patients with schizophrenia account for the largest proportion of inpatient cases (22, 23), and their quality of life has attracted the attention and research of many scholars (24–26), but further investigation is needed to explore the relationship between self-stigma and quality of life.

Therefore, the aims of this study are 1) to investigate the relationship between self-stigma and quality of life in patients with chronic schizophrenia, particularly which factors of self-stigma have a greater impact on quality of life, and 2) to explore the influencing factors of self-stigma and whether these factors affect quality of life via self-stigma. We hypothesize that all factors of self-stigma are related to quality of life and that the factors influencing self-stigma indirectly or directly affect quality of life via self-stigma. We plan to use multiple linear regression to investigate the influencing factors of self-stigma, and a mediation model will also be used to explore the mediating effect of self-stigma on quality of life.

## 2 Methods

### 2.1 Participants

This is a cross-sectional study based on hospitalized patients with schizophrenia. 170 inpatients with schizophrenia from Huilongguan Hospital in Beijing were selected as the subjects of the study using the random number table method. We conducted a study over a five-month period from June 2023 to October 2023, collecting data from patients at Beijing Huilonguan Hospital. All participants in the study were aged between 18 and 55 years. Additionally, every patient was capable of independently completing the questionnaire assessments without the need for assistance from medical staff. According to the WHO ICD-10 (27) criteria for the diagnosis of schizophrenia, all patients had been diagnosed with schizophrenia by at least two experienced psychiatrists. Eligible subjects were included in the study and written informed consent and demographic and clinical information were obtained from the patients. This study was approved by the Ethics Committee of Beijing Huilongguan Hospital. approved with grant number: 202324 section.

### 2.2 Inclusion and exclusion criteria

The inclusion criteria were as follows: (1) patients diagnosed with schizophrenia according to ICD-10; (2) stable medication use for at least 2 months; (3) aged between 18 and 55 years; (4) education level of junior high school or above; and (5) illness duration of ≥ 5 years. The exclusion criteria were as follows: (1) patients with major physical illnesses; (2) patients with organic brain disorders or a history of head trauma, significant intellectual disability, or other severe, uncontrollable physical illnesses; and (3) patients with psychiatric disorders caused by substance abuse such as alcoholism or drug addiction.

### 2.3 Psychopathological assessment

All participants were assessed using questionnaires, which were administered by two experienced psychiatrists. The intraclass correlation coefficient (ICC) indicated good consistency between raters (ICC = 0.80) in terms of rating scales.

#### 2.3.1 Positive and negative syndrome scale

The PANSS (28) scale is widely used to assess three main dimensions: positive syndrome (PANSS-P), negative syndrome (PANSS-N), and general psychopathology (GP). The values range from 1 (“absent”) to 7 (“extreme”). The scale’s total score ranges between 30 and 210 points, with higher scores indicating more severe symptoms. It serves as a tool for gauging the severity of symptoms in study participants. The internal consistency was good (Cronbach’s α>0.70) (29).

#### 2.3.2 Self-esteem inventory

The SEI (30) scale has a total of 58 questions, using a two-point scoring method: 1 point for being like me and 0 for not being like me. There are 30 questions in reverse scoring, The total score ranges from 0 to 50, and the higher the total score is, the higher the self-esteem level. The internal consistency coefficient of Cronbach Alpha of SEI was 0.86 (31).

#### 2.3.3 Connor-Davidson resilience scale

The CD-RISC (32) consists of 25 items rated on a 5-point similar scale, ranging from 0 (“not true at all”) to 4 (“true almost all of the time”). The total score ranges from 0 to 100, with higher scores indicating greater resilience. Cronbach’s alpha was 0.92 (33).

#### 2.3.4 Internalized stigma of mental illness

The ISMI (34) Scale, which contains 29 items distributed among five subscales that capture various aspects of the subjective experience of stigma: alienation, stereotype endorsement, perceived discrimination, social withdrawal and stigma resistance. These items are assessed on a 4-point scale, from 1 to 4, yielding a total possible score range of 29 to 116. The higher the score is, the greater the internalized stigma. Cronbach’s α coefficient is 0.94 (35).

#### 2.3.5 Schizophrenia quality of life scale

The SQLS (36) has a total of 30 items, which are divided into three subscales to evaluate psychosocial factors, motivation and energy, symptoms and side effects, respectively, using a 5-point scale (0-4). Each scale is scored from 0-100, and the higher the score, the worse the quality of life. Cronbach’s α coefficient is 0.83 -0.72 (37).

#### 2.3.6 Coping questionnaire for schizophrenic patients

The CQSP (38) scale has 54 items, and the questionnaire includes four subscales: “Problem Solving”, “Avoidance”, “Cognitive Adjustment” and “Emotion Regulation”. The sum of the item scores of each subscale is the total score of the subscale, and the higher the score.

A high score indicates that the coping style is more used to it. The questionnaire uses a 5-point Likert scale. The subjects had to choose each coping method (item) according to their own situation (1: I never use it, 2: I use it occasionally, 3: I use it more often, 4: I use it often, 5: I always use it). Cronbach’s α coefficient is 0.68 -0.95 (39).

#### 2.3.7 Social support rating scale

The SSRS (40) scale was used to measure the social support level of the participants (Cronbach α coefficient 0.80, P<0.001). The SSRS has 10 items in total, which are divided into three dimensions: “objective support”, “subjective support”, and “support utilization”. The total score is the sum of the scores of all ten items. The higher the score, the higher the social support level. A total score that is at most 22 is classified as a low level of social support, a score between 23 and 44 is classified as a medium level, and a score between 45 and 66 is classified as a high level. Cronbach’s α coefficient is 0.63 (41).

### 2.4 Statistical analysis

Statistical analyses were conducted following CONSORT guidelines and were performed using SPSS 22.0 software (SPSS, Chicago, IL, USA). The applied statistical methods were descriptive statistics for the demographic data, mean scores, and normality tests. First, we analyzed the basic information using descriptive statistics, which included percentages, mean values, and standard deviations. It is particularly important to emphasize that, to ensure consistency in dosage calculations, all medications taken by patients for the treatment of schizophrenia were converted into equivalent doses of olanzapine (42). Next, differences between scores were calculated by parametric or nonparametric paired or unpaired t tests or Kruskal–Wallis tests. We utilized regression analysis to predict the impact of the total self-stigma score and its respective factors on quality of life while also conducting correlation analysis to assess their associations with the total score and factors of quality of life. Finally, effect sizes were interpreted by Cohen. Citation51 A mediation analysis was performed by using the maximum likelihood method with standardized estimates. We performed mediation analysis to analyze the effects of self-esteem, coping strategies (1, 2), self-stigma, and quality of life. Direct and indirect effects were utilized to elucidate the relationship among these variables. Differences were significant when P values were less than 0.05.

## 3 Results

### 3.1 Demographic data

Out of 182 reviewed inpatients with schizophrenia, 170 were included in the study. The mean age of the study population was 51.46 years. Among the study population, 67.7% (115) were male and 33.3% (55) were female, with a mean age of 51.46 ± 12.52. Other demographic data, such as length of hospitalization and educational level, as well as clinical symptom-related assessments (including PANSS, SEI, CD-RISC, ISMI, SQLS, CQSP, SSRS), are shown in **Table 1**.

**Table 1:** Description of basic subject information.

Category	Figures or mean values
Number of patients	170
Age	51.46 ± 12.52
Sex: men/women	115/55
Education Year	12.02 ± 2.91
Course	22.00 ± 13.04
Onset	29.31 ± 10.32
Drug dosage	10.80 ± 6.68
PANSS-P	19.15 ± 3.36
PANSS-N	19.58 ± 4.50
PANSS-G	37.33 ± 9.32
PANSS-Total score	76.06 ± 9.32
SSRS-O	4.84 ± 2.82
SSRS-S	9.41 ± 2.53
SSRS-U	6.96 ± 2.31
SSRS-Total score	21.21 ± 5.68
CD-RISC-F1	19.14 ± 6.17
CD-RISC-F2	15.24 ± 4.56
CD-RISC-F3	12.46 ± 4.25
CD-RISC-F4	6.84 ± 2.94
CD-RISC-F5	4.43 ± 1.65
CD-RISC-Total score	58.10 ± 16.27
ISMI-A	20.86 ± 5.66
ISMI-SE	16.25 ± 4.64
ISMI-PD	11.40 ± 3.163
ISMI-SW	6.95 ± 2.45
ISMI-SR	11.12 ± 3.22
ISMI-Total score	66.58 ± 16.94
SQLS-P	20.79 ± 11.49
SQLS-ME	11.05 ± 4.07
SQLS-SSE	9.42 ± 5.90
SQLS-Total score	41.26 ± 18.67
CQSP-PS	67.13 ± 17.84
CQSP-A	37.08 ± 10.27
CQSP-CA	31.24 ± 8.82
CQSP-ER	19.5 ± 5.03
CQSP-Total score	154.94 ± 35.04
SEI-Total score	35.05 ± 7.68

PANSS, Positive and Negative Syndrome Scale; PANSS-P, Positive and Negative Syndrome Scale (Positive syndrome); PANSS-N, Positive and Negative Syndrome Scale (Negative syndrome); PANSS-G, Positive and Negative Syndrome Scale (General psychopathology); SSRS, Social Skills Rating Scale; SSRS-O, Social Skills Rating Scale (Objective support) ; SSRS-S, Social Skills Rating Scale (Subjective support) ; SSRS-U, Social Skills Rating Scale (Utilization of support);CD-RISC, Connor- Davidson resilience scale; CD-RISC-F1, Connor-Davidson Resilience Scale (Adversity); CD-RISC-F2, Connor-Davidson Resilience Scale (Persistence); CD-RISC-F3, Connor-Davidson Resilience Scale (Adaptability); CD-RISC-F4, Connor-Davidson Resilience Scale (Positive Acceptance); CD-RISC-F5, Connor-Davidson Resilience Scale (Secure Relationships); SQLS, Schizophrenia Quality of Life Scale; SQLS-P, Schizophrenia Quality of Life Scale (Psychosocial); SQLS-ME, Schizophrenia Quality of Life Scale (Motivation and energy); SQLS- (SSE), Schizophrenia Quality of Life Scale (Symptoms and side Effects); ISMI, Internalized Stigma of Mental Illness; ISMI-A, Internalized Stigma of Mental Illness (Alienation); ISMI-SE, Internalized Stigma of Mental Illness (Stereotype Endorsement); ISMI-PD, Internalized Stigma of Mental Illness (Perceived Discrimination); ISMI-SW, Internalized Stigma of Mental Illness (Social Withdrawal); ISMI-SR, Internalized Stigma of Mental Illness (Stigma Resistance); CQSP, Coping Questionnaire for Schizophrenic Patients; CQSP-PS, Coping Questionnaire for Schizophrenic Patients (Problem Solving); CQSP-A, Coping Questionnaire for Schizophrenic Patients (Avoidance); CQSP-CA, Coping Questionnaire for Schizophrenic Patients (Cognitive Adjustment); CQSP-ER, Coping Questionnaire for Schizophrenic Patients (Emotion Regulation); SEI, Self-esteem Inventory.

### 3.2 Prediction of total self-stigma score on overall quality of life (regression analysis)

The results showed a significant impact of the total self-stigma score on quality of life (F = 12.56, P = 0.000^b^, Beat = 0.56, T = 8.13, *p* = 0.00).

### 3.3 Correlation analysis between total and subscores of self-stigma and quality of life

The results showed that there were significant correlations between the total and subscores of self-stigma and the total and subscores of quality of life. See **Table 2** for details. Additionally, we conducted a correlation analysis between self-stigma and sociodemographic characteristics; however, no significant results were identified.

**Table 2:** Correlation analysis between self-stigma score and its factors and quality of life score and factors.

	SQLS-P	SQLS-ME	SQLS-SSE	SQLS-Total score	ISMI-A	ISMI-SE	ISMI-PD	ISMI-SW	ISMI-SR	ISMI-Total score
SQLS-P	1									
SQLS-ME	0.39**	1								
SQLS-SSE	0.80**	0.41**	1							
SQLS-Total score	0.96**	0.60**	0.90**	1						
ISMI-A	0.54**	0.28**	0.47**	0.54**	1					
ISMI-SE	0.53**	0.29**	0.47**	0.54**	0.82**	1				
ISMI-PD	0.51**	0.27**	0.50**	0.53**	0.71**	0.73**	1			
ISMI-SW	0.44**	0.19*	0.35**	0.43**	0.70**	0.70**	0.62**	1		
ISMI-SR	0.46**	0.21**	0.41**	0.46**	0.78**	0.79**	0.72**	0.69**	1	
ISMI-Total score	0.57**	0.29**	0.50**	0.57**	0.93**	0.93**	0.85**	0.81**	0.89**	1

Note: *P<0.05, **P<0.01. SQLS, Schizophrenia Quality of Life Scale; ISMI, Internalized Stigma of Mental Illness; SQLS-P, Schizophrenia Quality of Life Scale(Psychosocial); SQLS-ME, Schizophrenia Quality of Life Scale(Motivation and energy); SQLS-(SSE), Schizophrenia Quality of Life Scale(Symptoms and side Effects); ISMI-A, Internalized Stigma of Mental Illness(Alienation); ISMI-SE, Internalized Stigma of Mental Illness(Stereotype Endorsement); ISMI-PD, Internalized Stigma of Mental Illness (Perceived Discrimination); ISMI-SW, Internalized Stigma of Mental Illness (Social Withdrawal); ISMI-SR, Internalized Stigma of Mental Illness (Stigma Resistance).

### 3.4 Regression analysis of self-stigma factors on overall quality of life score

The results showed that perceived discrimination (T = 2.22, *p* = 0.03) and stigma resistance (T = 2.65, *p* = 0.01) in the self-stigma factors had a significant impact on quality of life, while the other self-stigma factors had no significant impact on the overall quality of life score. See **Table 3** for details (R^2^ = 0.37, Adjusted R^2^ = 0.35).

**Table 3:** The regression analysis of self-stigma factors for total quality of life scores.

Dependent Variable	Predictors	Beta	T	P
SQLS (F = 8.99) (P = 0.000^b^)	ISMI-A	0.17	1.39	0.17
	ISMI-SE	0.28	2.22	0.03*
	ISMI-PD	0.27	2.65	0.01*
	ISMI-SW	-0.02	-0.17	0.87
	ISMI-SR	-0.08	-0.65	0.51

SQLS, Schizophrenia Quality of Life Scale; ISMI, Internalized Stigma of Mental Illness; ISMI-A, Internalized Stigma of Mental Illness (Alienation); ISMI-SE, Internalized Stigma of Mental Illness (Stereotype Endorsement); ISMI-PD, Internalized Stigma of Mental Illness (Perceived Discrimination); ISMI-SW, Internalized Stigma of Mental Illness (Social Withdrawal); ISMI-SR, Internalized Stigma of Mental Illness (Stigma Resistance). * p < 0.05.

### 3.5 Factors influencing self-stigma

The relationship between various factors in the PANSS, SEI, CQSP, SSRS, and CD-RISC and self-stigma was explored. The results showed that self-esteem (T = -2.11, *p* = 0.04), coping style 1 (problem-solving factor) (T = -2.58, *p* = 0.01), and coping style 2 (avoidance factor) (T = 4.65, *p* = 0.00) had a greater impact on self-stigma. See **Table 4** for details (R^2^ = 0.46, Adjusted R^2^ = 0.42).

**Table 4:** Explore which factors have the greatest influence on self-stigma.

Dependent Variable	Predictors	Beta	T	P
ISMI (F = 5.17) (P = 0.000^b^)	PANSS-P	-0.13	-1.90	0.06
	PANSS-N	0.08	1.26	0.21
	PANSS-G	-0.10	-1.46	0.15
	SEI	-0.17	-2.11	0.04*
	CQSP-PS	-0.44	-2.58	0.01*
	CQSP-A	0.39	4.65	0.00*
	CQSP-CA	0.17	1.07	0.29
	CQSP-ER	0.14	1.34	0.18
	SSRS-O	-0.05	-0.70	0.49
	SSRS-S	0.02	0.19	0.85
	SSRS-U	-0.02	-0.24	0.81
	CD-RISC-F1	0.10	0.86	0.40
	CD-RISC-F2	0.06	0.51	0.61
	CD-RISC-F3	-0.05	-0.52	0.60
	CD-RISC-F4	0.04	0.48	0.63
	CD-RISC-F5	-0.10	-1.18	0.24

ISMI, Internalized Stigma of Mental Illness; PANSS-P, Positive and Negative Syndrome Scale (Positive syndrome ); PANSS-N, Positive and Negative Syndrome Scale (Negative syndrome); PANSS-G, Positive and Negative Syndrome Scale (General psychopathology); SEI, Self-esteem Inventor; CQSP-PS, Coping Questionnaire for Schizophrenic Patients (Problem Solving); CQSP-A, Coping Questionnaire for Schizophrenic Patients (Avoidance); CQSP-CA, Coping Questionnaire for Schizophrenic Patients (Cognitive Adjustment); CQSP-ER, Coping Questionnaire for Schizophrenic Patients (Emotion Regulation); SSSRS-O, Social Skills Rating Scale (Objective support) ; SSRS-S, Social Skills Rating Scale (Subjective support) ; SSRS-U, Social Skills Rating Scale (Utilization of support); CD-RISC-F1, Connor-Davidson Resilience Scale (Adversity); CD-RISC-F2, Connor-Davidson Resilience Scale (Persistence); CD-RISC-F3, Connor-Davidson Resilience Scale (Adaptability); CD-RISC-F4, Connor-Davidson Resilience Scale (Positive Acceptance); CD-RISC-F5, Connor-Davidson Resilience Scale (Secure Relationships). * p < 0.05.

### 3.6 Exploring whether these three factors (self-esteem, problem-solving coping style, and avoidance coping style) affect quality of life via self-stigma

**Figure 1:**
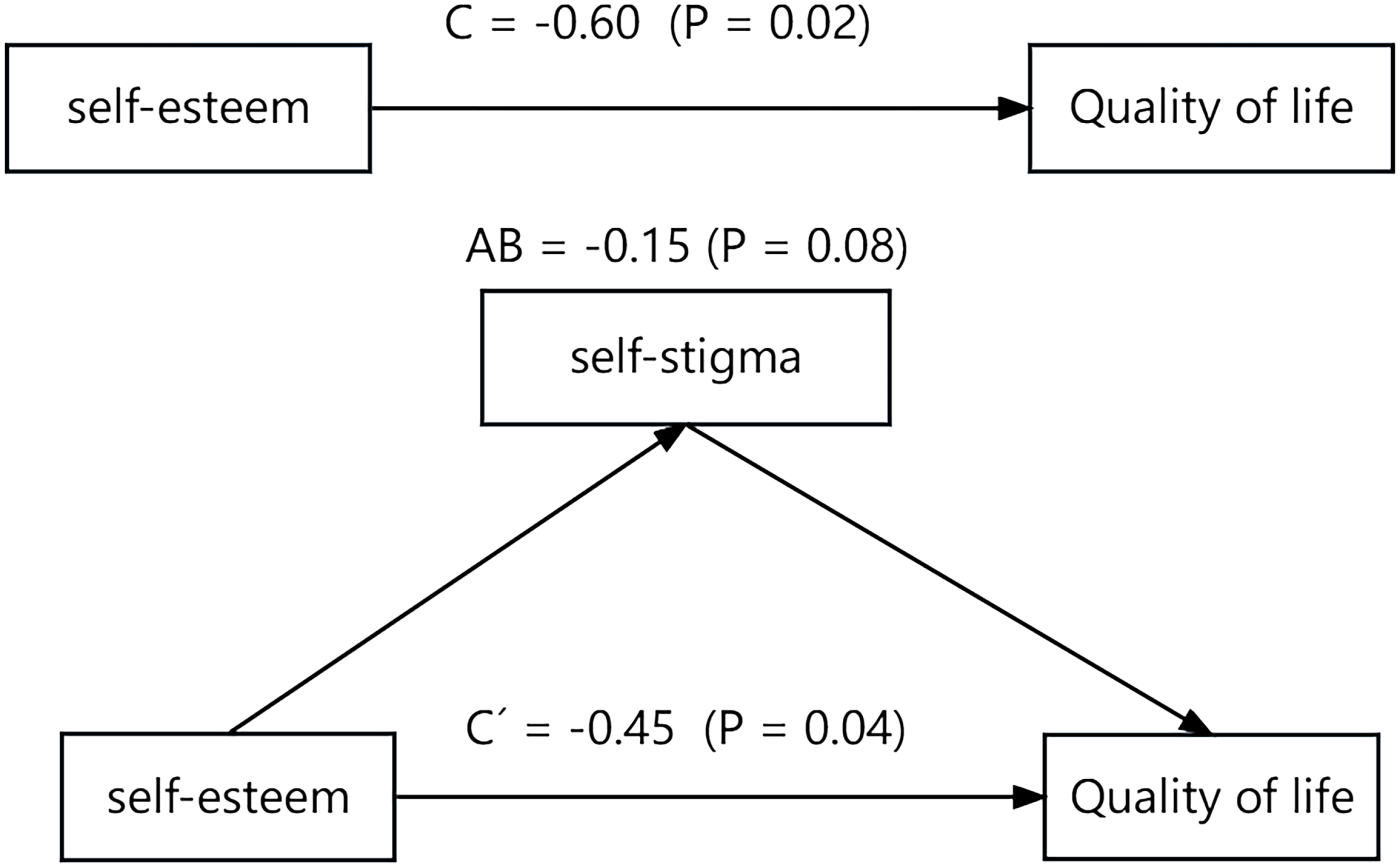
The mediating role of self-stigma in the relationship between self-esteem and quality of life.

Mediation analysis was conducted to examine the relationships between self-esteem, coping strategies (problem-solving and avoidance), self-stigma, and quality of life. The results revealed that self-esteem had a significant effect on quality of life (C´ = -0.45, *p* = 0.04), as did self-stigma (C = -0.60, *p* = 0.02). However, the indirect effect of self-esteem on quality of life via self-stigma was not significant (AB = -0.15, *p* = 0.08). Further details can be found in **Figure 1**.

**Figure 2:**
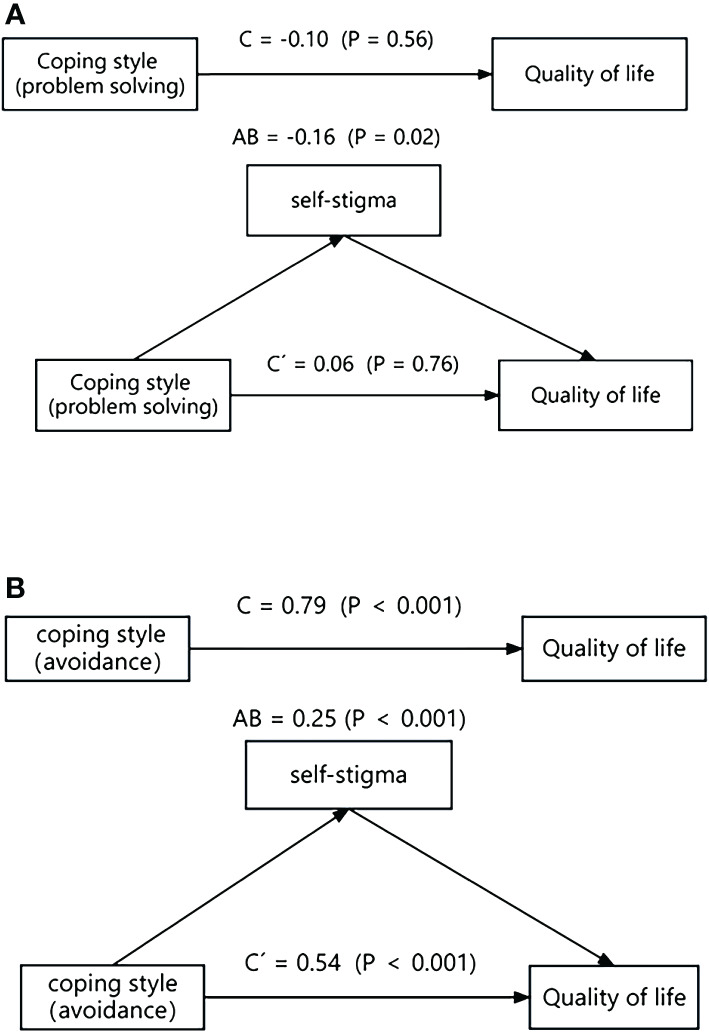
The mediating role of self-stigma in the relationship coping styles and quality of life.

On the other hand, the analysis showed a significant indirect effect of problem-solving coping style on quality of life via self-stigma (AB = -0.16, *p* = 0.02). This suggests that the problem-solving coping style influences quality of life by affecting self-stigma. Additionally, the results indicated that the avoidance coping style had both significant direct effects (C’ = 0.54, *p* < 0.001) and significant indirect effects (AB = 0.25, *p* < 0.001) on quality of life via self-stigma. This suggests a partial mediation effect, where lower avoidance scores are associated with lower self-stigma and higher quality of life. Further details can be found in **Figure 2**.

## 4 Discussion

The main results of this study are as follows: First, self-stigma significantly impacts quality of life, with perceived discriminations and stigma resistance within the self-stigma factors significantly affecting quality of life. Second, self-esteem, problem-solving coping style, and avoidance coping style have a significant influence on self-stigma. Last, problem-solving coping style (Factor 1) exerts an effect on quality of life via self-stigma, with a significant mediating effect. Similarly, avoidance coping style (Factor 2) affects quality of life via self-stigma, also showing a significant mediating effect.

This study found a significant impact of self-stigma on the quality of life of individuals with schizophrenia, with perceived discriminations and stigma resistance being significant factors within the self-stigma construct. In a study examining self-stigma, sleep quality, and quality of life among individuals with mental disorders, it was found that self-stigma influences quality of life by affecting sleep quality (43). A multinational study conducted in the Americas revealed a significant impact of self-stigma on quality of life (44). A cross-sectional survey involving 153 individuals with mental disorders showed that among the various psychological or social influences on quality of life, self-stigma emerged as the most significant factor (45). Another study that included a systematic review of 63 articles (N = 8925, 22 countries) and a meta-analysis of 53 articles (N = 7756) revealed a strong negative correlation between self-stigma and quality of life (46). Many studies have shown that a significant factor contributing to self-stigma is public stigma (47–49), and stigma pressure from society leads to social withdrawal among individuals with mental illness (50, 51).

Which factors influence self-stigma? This study found that among the various factors explored, self-esteem, problem-solving coping style, and avoidance coping style have significant effects on self-stigma. Sarraf et al.’s study found a significant impact of self-esteem on self-stigma in social psychological variables (47). Additionally, Jian et al. discovered that self-esteem moderates the association between self-stigma and suicide risk, indicating that enhancing self-esteem can effectively reduce self-stigma (52). Coping styles are frequently discussed in the field of mental illness, and research has shown significant associations between self-stigma, illness severity, and coping strategies in individuals with schizophrenia. The use of negative coping strategies increases self-stigma in individuals with schizophrenia (45). Moreover, avoidance coping is negatively correlated with shame resistance, and reducing avoidance coping behaviors can effectively lower self-stigma (45, 53, 54). Our study’s findings align with the aforementioned research results. It is noteworthy that in a study on self-stigmatization among patients with schizophrenia, it was highlighted that the side effects of medication significantly impact self-stigma (55). Additionally, other literature indicates a negative correlation between subjective well-being and self-stigma, the stronger the sense of subjective well-being, the lesser the self-stigma experienced (56, 57). In clinical treatment, focusing on self-esteem, coping styles, medication side effects, and subjective well-being is crucial for intervening in self-stigma.

Based on the two aforementioned conclusions, we also conducted an analysis on whether self-esteem, problem-solving coping style, and avoidance coping style have an indirect impact on quality of life via self-stigma. First, the research findings indicate that the problem-solving coping style affects quality of life via self-stigma, with a significant full mediating effect. However, the problem-solving coping style does not have a direct impact on quality of life; its influence is primarily mediated by self-stigma. Holubova et al.’s study revealed that quality of life is primarily influenced by self-stigma and negative coping styles. Using structural equation modeling, they analyzed the interrelationships between these factors and found that self-stigma serves as the main contributing factor (45). From a reverse mediation perspective, our research results are consistent with numerous previous studies, indicating that higher levels of problem solving are associated with lower self-stigma and higher quality of life (19, 54, 58).

Next, another result shows that avoidant coping strategies can directly impact quality of life and, at the same time, partially influence it via self-stigma. There is a reciprocal causal relationship between avoidance and self-stigma, forming a vicious cycle (59, 60). Cavelti et al.’s study also demonstrated that lower scores on avoidance are associated with lower self-stigma and higher quality of life (61). Therefore, we have found that there are many factors influencing the quality of life of individuals with schizophrenia, and it is a complex process. Self-stigma is an important factor that not only directly affects quality of life but also serves as a mediator influencing quality of life.

According to the World Health Organization’s 2017 Mental Health Atlas, the proportion of new admissions to long-term hospitalizations (1 year or longer) has decreased to approximately 12% (62). However, over two-thirds of psychiatric patients have an overall hospitalization duration exceeding 1 year (63). Some studies suggest that self-stigma may be one of the reasons for long-term hospitalization (26, 64). Our research findings indicate that self-stigma significantly affects quality of life, with self-esteem and coping strategies—particularly problem-solving and avoidance—having a significant impact on self-stigma. This aligns with the findings of Huang et al., where self-esteem plays an important mediating role between self-stigma and quality of life (65). A cross-sectional study also revealed the relationship among self-stigma, coping methods, and quality of life (45).

Based on these findings, it is suggested that clinical interventions should focus on reducing self-stigma, particularly by enhancing self-esteem and promoting adaptive coping strategies. Moreover, strategies worth considering include reducing public stigma (66), targeted psychoeducation (67), cognitive restructuring techniques in cognitive-behavioral therapy, and increasing well-being (68–70), all aimed at improving self-stigma and quality of life for long-term hospitalized patients.

## 5 Limitations

There are several limitations to this study. First, the cross-sectional design of this study has inherent general and specific limitations. As our patients were evaluated at only one time point, the accuracy over time cannot be ensured. Second, the participants were all from the same psychiatric hospital in Beijing, China, which limits the generalizability of the study to the entire Chinese mainland. The theoretical model may change or expand if participants from other cities, regions, countries, or ethnic groups are included. In addition, the sample size of this study was only 170 individuals with schizophrenia, which may have limited the generalizability of the research findings.

## 6 Conclusion

The self-stigma experienced by individuals with schizophrenia significantly impacts their quality of life. Common factors influencing self-stigma are self-esteem and coping strategies. Both self-esteem and coping strategies directly or indirectly affect quality of life via self-stigma. We recommend that clinical efforts to improve the quality of life for individuals with schizophrenia should focus on self-stigma and its subfactors, particularly by addressing the mediating role of self-stigma and examining the impact of self-esteem and coping strategies on quality of life. Strategies such as enhancing self-esteem and modifying communication styles can be employed to reduce self-stigma and enhance quality of life.

## 7 Future research directions

This study has discussed the relationship between self-stigma and quality of life in patients with schizophrenia, as well as the mediating role of self-stigma. It is well-known that schizophrenia frequently co-occurs with other mental disorders or certain psychiatric symptoms. Research on autism symptoms in schizophrenia—specifically, their effects on internalized shame, well-being, and clinical and functional characteristics—suggests that symptoms of autism spectrum disorders may act protectively against self-stigma. Therefore, future research could more broadly investigate the impacts of self-stigma, considering the comorbidity of schizophrenia with other conditions.

## Data availability statement

The original contributions presented in the study are included in the article/supplementary material, further inquiries can be directed to the corresponding author/s.

## Ethics statement

This study involving human participants was reviewed and approved by the Ethics Committee of Beijing Huilongguan Hospital, with the approval number: 202324. The patients/participants provided their written informed consent to participate in this study.

## Author contributions

FL: Data curation, Methodology, Resources, Writing – original draft. HD: Data curation, Formal analysis, Writing – review & editing. NH: Conceptualization, Project administration, Validation, Visualization, Writing – original draft. WH: Formal analysis, Validation, Writing – review & editing. HW: Investigation, Software, Supervision, Validation, Writing – original draft. LL: Funding acquisition, Methodology, Supervision, Writing – review & editing. JC: Conceptualization, Data curation, Formal analysis, Investigation, Writing – review & editing. YL: Conceptualization, Data curation, Formal analysis, Funding acquisition, Investigation, Methodology, Project administration, Resources, Software, Supervision, Validation, Visualization, Writing – original draft, Writing – review & editing.
